# Structural basis for the distinct roles of non-conserved Pro116 and conserved Tyr124 of BCH domain of yeast p50RhoGAP

**DOI:** 10.1007/s00018-024-05238-8

**Published:** 2024-05-13

**Authors:** Srihari Shankar, Ti Weng Chew, Vishnu Priyanka Reddy Chichili, Boon Chuan Low, J. Sivaraman

**Affiliations:** 1 Department of Biological Sciences, National University of Singapore, 14 Science Drive 4, Singapore, 117543 Singapore; 2 Mechanobiology Institute, National University of Singapore, Singapore, 117411 Singapore; 3 NUS College, National University of Singapore, Singapore, 138593 Singapore

**Keywords:** BCH domain, GTPase-activating protein, Rho, Scaffold, Structure

## Abstract

p50RhoGAP is a key protein that interacts with and downregulates the small GTPase RhoA. p50RhoGAP is a multifunctional protein containing the BNIP-2 and Cdc42GAP Homology (BCH) domain that facilitates protein–protein interactions and lipid binding and the GAP domain that regulates active RhoA population. We recently solved the structure of the BCH domain from yeast p50RhoGAP (_Y_BCH) and showed that it maintains the adjacent GAP domain in an auto-inhibited state through the β5 strand. Our previous WT _Y_BCH structure shows that a unique kink at position 116 thought to be made by a proline residue between alpha helices α6 and α7 is essential for the formation of intertwined dimer from asymmetric monomers. Here we sought to establish the role and impact of this Pro116. However, the kink persists in the structure of P116A mutant _Y_BCH domain, suggesting that the scaffold is not dictated by the proline residue at this position. We further identified Tyr124 (or Tyr188 in _H_BCH) as a conserved residue in the crucial β5 strand. Extending to the human ortholog, when substituted to acidic residues, Tyr188D or Tyr188E, we observed an increase in RhoA binding and self-dimerization, indicative of a loss of inhibition of the GAP domain by the BCH domain. These results point to distinct roles and impact of the non-conserved and conserved amino acid positions in regulating the structural and functional complexity of the BCH domain.

## Introduction

Cell signalling is mediated by complex, intricate networks of proteins that regulate many cellular processes. GTPases are one important component of these networks. In response to localised cues within the cell, GTPases are activated through binding to GTP via the activities of guanine nucleotide exchange factors (GEFs) and deactivated by the hydrolysis of the GTP to GDP [1] with the help of GTPase-activating proteins (GAPs) [2, 3]. Lipid binding and other post-translational modifications (PTM) modulate GAP and GEF functions; however, their precise mechanisms of action remain largely unknown. The proteins responsible for bringing GEFs and GAPs to target proteins crucially determine the rate of RhoA inactivation by GAPs, a signalling mechanism that is also relatively less well described [2, 4, 5] when compared to its catalysis mechanism that is well understood.

The BNIP-2 and Cdc42GAP Homology (BCH) domain is present in 175 homologous proteins [29] (such as BNIP-2, p50RhoGAP and BPGAP1) and regulates various key cellular processes. BCH domain-containing proteins associate with many target proteins such as RhoA, Cdc42, Kidney-type Glutaminase (KGA), Kinesin, Pin1, Ras, GEFs and LATS/Hippo to regulate apoptosis, cell motility, cell signalling, cell protrusions, neuritogenesis and cardiomyoblast differentiation [6–14]. Some BCH family members, such as p50RhoGAP, contain additional domains such as the GAP domain, [15] which are crucial in determining cell fate and function through their involvement in signalling pathways [2, 16, 17].

There are various isoforms of the small GTPases, such as Rac and Rho, the dysregulations of which have been linked with distinct functions in diseases and disorders [3, 18–20]. Through their interactions with the Rho or Rac subfamily members, the BCH and GAP domains work in concert to regulate the population of these active small GTPases [4, 21]. The catalytic arginine in the GAP domain is responsible for maintaining the substrate GTPase in a stable transition state before carrying out GTP hydrolysis [22].

In general, substrate specificity of the BCH domain is dictated by well-studied “switch regions” on the substrate, with protein conformation relying on the type of nucleotide that is engaged in binding with the substrate [23–25]. Additional, smaller, non-switch regions and amino acid sequence variations further refine these preferences for specific isoforms [21, 25, 26]. Posttranslational modifications to small GTPases not only effectively regulate their spatial distribution, but also help them carry out favourable conformational changes that will permit binding with substrates. Lipid additions, such as prenylation, contribute to functional specialization and membrane localization and are primarily observed in Rho and Rac small GTPases [27–29]. Lastly, regulation mechanisms exist in proteins that contain the GAP domain: auto-inhibition by the BCH domain, which is highly influenced by the addition of a lipid moiety, was predicted to be a regulatory mechanism in the p50RhoGAP [29, 30]. We recently reported the crystal structure of the novel BCH domain which shed light on the molecular mechanism behind this auto-inhibitory regulation in addition to its binding to Rho [31].

Here, we show that, compared with the human BCH domain, the Proline in position 116 is less conserved whereas Tyrosine in position 124 is highly conserved in the yeast ortholog. Pro116 is located at a structurally important kink region in the BCH domain of *S.* *pombe* p50RhoGAP (henceforth called yeast BCH or _Y_BCH). Therefore, we determined the structure of the mutant P116A _Y_BCH but found that no differences exist between the mutant and WT _Y_BCH structures. Through cell-based assays, we further showed that this Proline substitution has no significant effect on the functionality of p50RhoGAP. In contrast, there is a highly conserved Tyrosine at position Y188 in _H_BCH (equivalent to position 124 in β5 strand of yeast BCH or _Y_BCH), which is found mutated (as a Y188C mutation) in ovarian cancer [32]; the functional impact of this is not known. Y188 in _H_BCH (or 124 _Y_BCH) is in an important secondary structural element that maintains the GAP domain of p50RhoGAP in an auto-inhibitory conformation [31]. Our mutational studies identified a putative role for Y188 in the control of autoinhibition between the GAP domain and the BCH domain.

## Results

### Sequence analysis revealed the presence of key non-conserved and conserved amino acids at positions 116 and 124, respectively

**Fig. 1:**
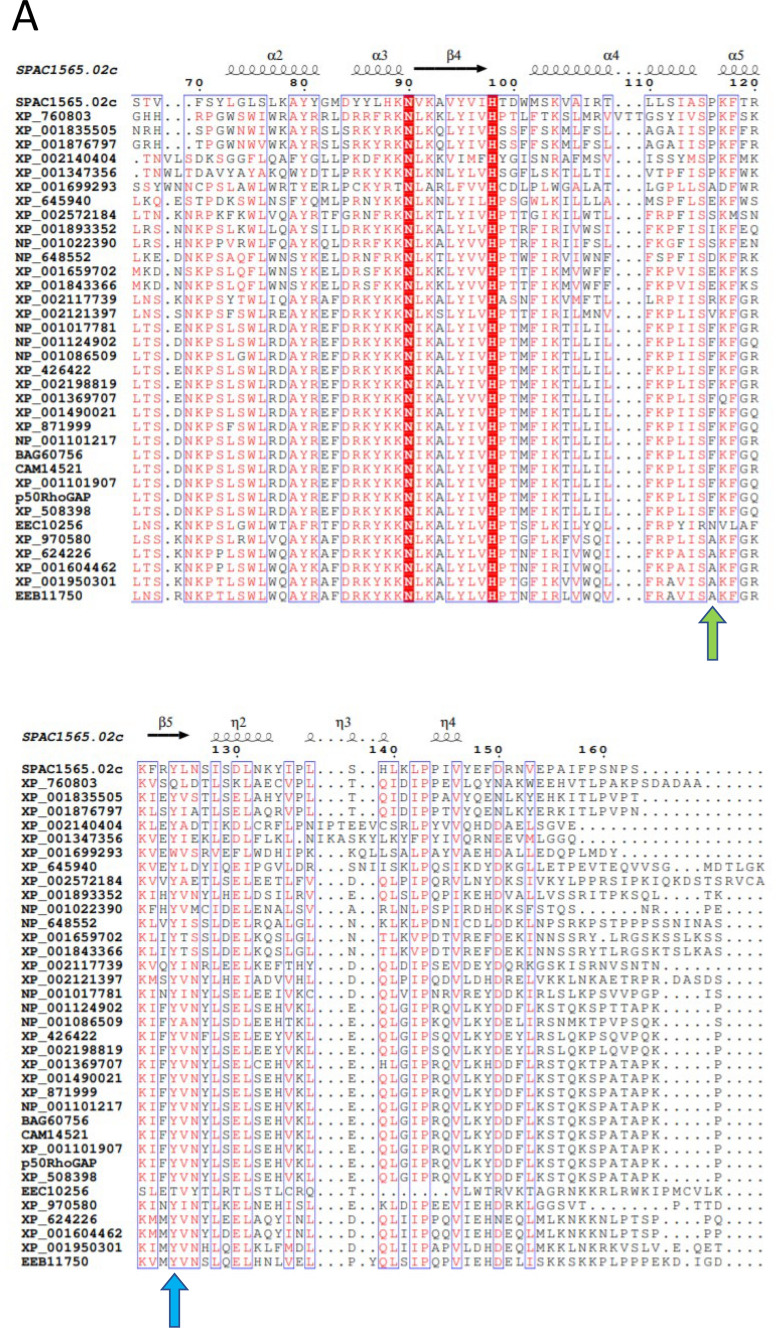

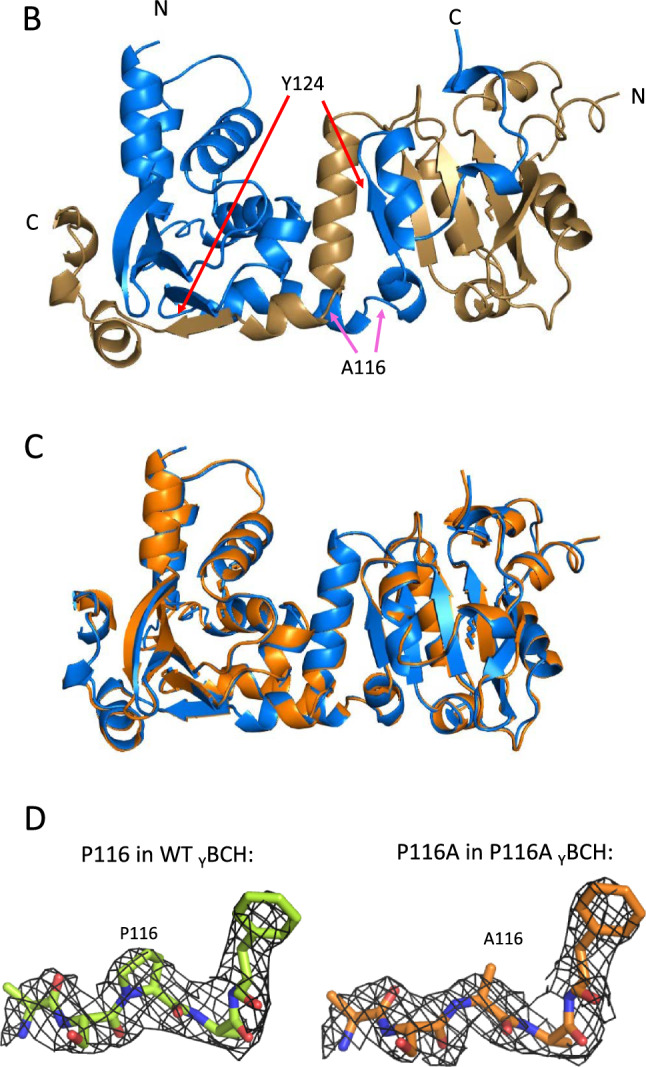
Sequence analysis and crystal structure of P116A mutant structure. **A** The sequence analysis of the 36 sequences shows conserved amino acids. The sequences were taken mainly from Group III which primarily consists of different species variants of p50RhoGAP. Additionally, two more groups, Group IIa and Group IIb were included in the analysis for a more extensive analysis. We find that the structurally important position 116 (green arrow) does not have a conserved amino acid. It contains Proline in the _Y_BCH and Phenylalanine in the _H_BCH. Alanine is also a popular amino acid found in this position. Despite these differences in amino acids, the structure remains the same between the wild-type and mutant structures thereby suggesting that the structures are representative of a larger family of BCH domain containing proteins. Additionally, position 124 (blue arrow) shows the conserved Tyrosine residue that is likely phosphorylated in the BCH domain of the p50RhoGAP. **B** The mutant structure comprises of a dimer of dimers in the asymmetric unit. The functional unit is the dimer which consists of asymmetric monomers that are intertwined with one another from Arg108. The Ala116 and Tyr124 as well as the N and C termini are labelled. **C** The structural superposition of the wild-type (orange) and mutant (blue) dimers show that they are well aligned. **D** The *2Fo-Fc* electron density map at 0.8 σ contour is shown for the regions 114–118 aa of the wild-type (PDB 7E0W) and P116A mutant _Y_BCH structures (PDB 8K70 from the current study). It shows that at position 116 of each structure there is clear density for the pyrrolidine ring of Proline (WT) and the methyl group of Alanine (P116A), respectively. These structures are of similar resolution (2.80 Å for 7E0W and 2.81 Å for 8K70).

We recently reported that the structure of the _Y_BCH domain contains two asymmetric monomers in the dimer, in which the monomers intertwine to form the dimeric BCH domain, with the direction of the polypeptide chain changing at Pro116 [31]. Given this feature, we sought to investigate the conserved nature of a proline residue in the α5 region of _Y_BCH domain among various isoforms across different species. The BCH domain is known to have evolutionarily variegated from the CRAL-TRIO domain into three classes [33]. The p50RhoGAP and its various species-specific isoforms constitute the group III BCH domain, which is closely related to the group IIa and group IIb classes. A total of 36 sequences from all the three classes (group III, IIa and IIb) were used in the sequence comparison **(**Fig. 1A**)**.

Sequence alignment shows non conserved residues at position 116 in the BCH domain, with phenylalanine accounting for 14 out of the 36 sequences (Fig. 1A), including the human sequence. Alanine and proline are present in 6 sequences each. Serine and glutamic acid substitutions occur less frequently. Further sequence analysis highlighted high conservation (33/36 sequences) of a tyrosine residue at position 124 in _Y_BCH (position 188 in _H_BCH). It is located in the β5 strand, which is a crucial secondary structural element involved in the functioning of p50RhoGAP [31].

### Structural conservation between P116A and WT _YBCH_ domain

To understand the structural role of Pro116, we created a P116A substitution mutant of the _Y_BCH domain, purified and crystallized this domain that diffracted up to 2.8 Å resolution (Table 1). Like the WT _Y_BCH domain, the crystal structure of the P116A mutant forms an intertwined dimer from two non-identical monomers mainly at the β5 region from Arg108 onwards (Fig. 1B).

**Table 1:** Crystallographic data and refinement statistic

	P116A mutant
Data collection	
Space group	P6_1_
Unit cell parameters (Å, º)	a = b = 108.11, c = 250.80, α = β = 90, γ = 120
Resolution range (Å)*	50.0–2.8 (2.87–2.81)
Wavelength (Å)	1.5418
Observed *hkl*	237401
Unique *hkl*	37961
Completeness (%)	93.83
Overall *I/*σ*I*	19.7
^a^Rsym (%)	14.2
Refinement and quality of the model	
Resolution range (Å)	49.64–2.81
^b^R_work_ (%)	0.21 (37961)
^c^R_free_ (%)	0.24 (2084)
rmsd bond length (Å)	0.011
rmsd bond angle (º)	1.27
^d^Ramachandran plot (%)	
Allowed regions	98.0
Disallowed regions	2.0

*Values in parentheses correspond to the highest resolution shell

*Rmsd* Refinement and quality of the model

^a^R_sym_ = ∑∑ | I (k)—<I>|/∑ I (k) where I (k) and <I> represent the diffraction intensity values of the individual measurements and the corresponding mean values. The summation is over all unique measurements.

^b^R_work_ = ∑ ||Fobs|—k|Fcalc||/|Fobs| where Fobs and Fcalc are the observed and calculated structure factors, respectively.

^c^R_free_ is the sum extended over a subset of reflections (5%) excluded from all refinement stages.

^d^As calculated using MolProbity .

The _Y_BCH P116A dimer superimposed with the _Y_BCH WT dimer with a rmsd of 0.16 Å for 299 Cα atoms. Monomers within the WT _Y_BCH dimer showed structural distinctions beyond Pro116, which was also observed in the P116A monomers in the _Y_BCH P116A dimer (Fig. 1C). Alanine in position 116 (P116A mutant) orients the polypeptide chain beyond position 116 in a manner similar to that of proline in the WT structure (Fig. 1D). Therefore, we identified no major structural change following proline to alanine substitution at position 116. Moreover, the hydrogen bonds and buried interface areas between the monomers of the dimer are largely similar between the WT and P116A mutant _Y_BCH structures (buried area 2861.5 Å [2] [WT] vs. 2882.3 Å [2] [P116A]), which suggests that the scaffold of the dimeric BCH domain maintains its structure and thus function irrespective of the amino acid at that location. Collectively, these results suggest that proline, although known to cause structural changes in other situations [34, 35], is not responsible for the kink in the polypeptide chain in *S. pombe* p50RhoGAP.

**Fig. 2:**
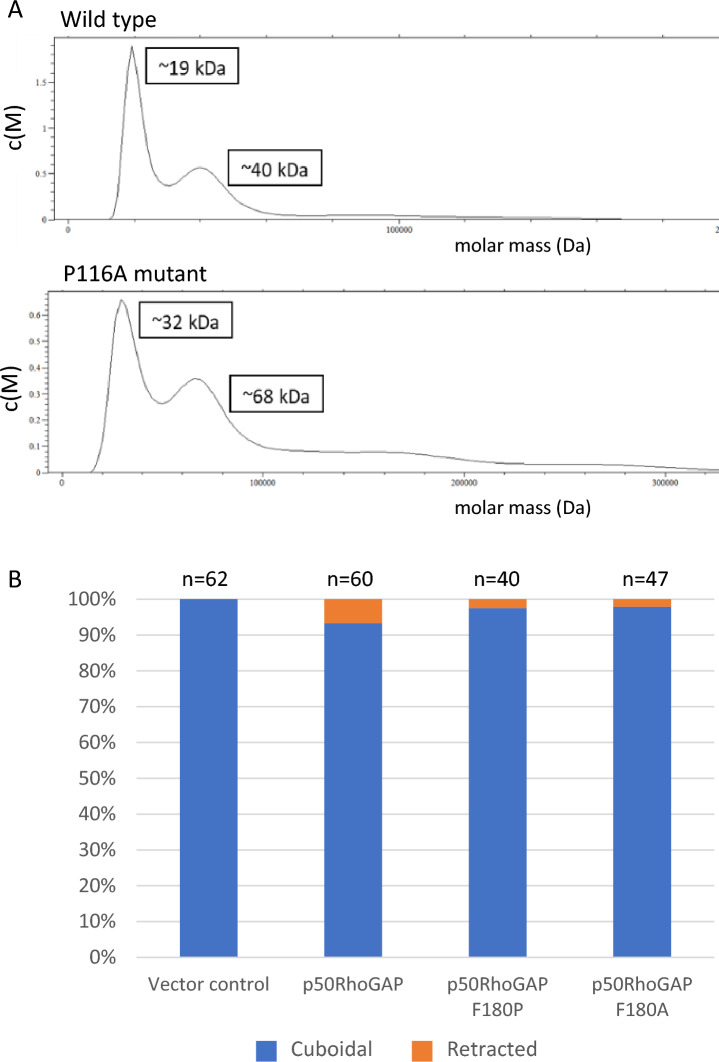
**A** The analytical ultracentrifuge results show that the wild-type protein mainly forms monomers and dimers while the mutant protein shows predominantly higher order oligomers such as dimers and tetramers. **B** HeLa JW cells were co-transfected with HA-RhoA and FLAG-tagged p50RhoGAP or its mutants as labelled. Cells were fixed and immunostaining was conducted to identify cells that incorporated both plasmids. Images were captured by W1 spinning disk confocal microscopy and scored for their morphologies

Next, we sought to assess the populations of different oligomeric forms of the _Y_BCH domain in solution at the crystallization concentration (2.4 mg/ml). This was achieved using analytical ultracentrifugation (AUC). We note that the WT _Y_BCH protein in AUC mainly exists as monomers, showing only 20% dimer (80% monomer) formation. Contrastingly, at the same concentration, the P116A mutant protein in AUC shows 70% dimers and 30% tetramers (dimer of dimers), indicating that higher order oligomerization occurs among the P116A mutant of _Y_BCH (Fig. 2A). In comparing the WT and P116 structures, however, no structural reasons could be provided to explain this difference. One possible explanation is that the tetramer is a more crystallizable form than a dimer. Notably, the oligomeric forms that we compare here, between the WT and P116A mutants, are at the same concentration in solution, and they crystallize under similar conditions. Despite their differences in oligomerization behavior in solution, the structure and function are the same. While oligomeric difference between the mutant and wild type is interesting, we do not have any explanation yet, and this will be investigated in future studies.

### Cell rounding assays show similar effects for wild-type and mutant human p50RhoGAP

The human ortholog of the _Y_BCH has a phenylalanine at position 180 (_H_BCH) whereas the yeast BCH has a proline at position 116. HeLa cells were transfected with WT FLAG-tagged p50RhoGAP and either of two mutants: F180P, resembling the yeast isoform, and F180A, an alanine substitute; alanine was chosen as it was the next most popular amino acid (Fig. 1A). Cell rounding assays were used to examine the GAP activity. The results showed largely similar effects among the WT and mutant proteins (Fig. 2B), suggesting that F180 is neither sufficient nor necessary in maintaining the intra-molecular inhibition important for controlling the GAP activity.

### Conserved tyrosine mediates the interaction between RhoA and p50RhoGAP

**Fig. 3:**
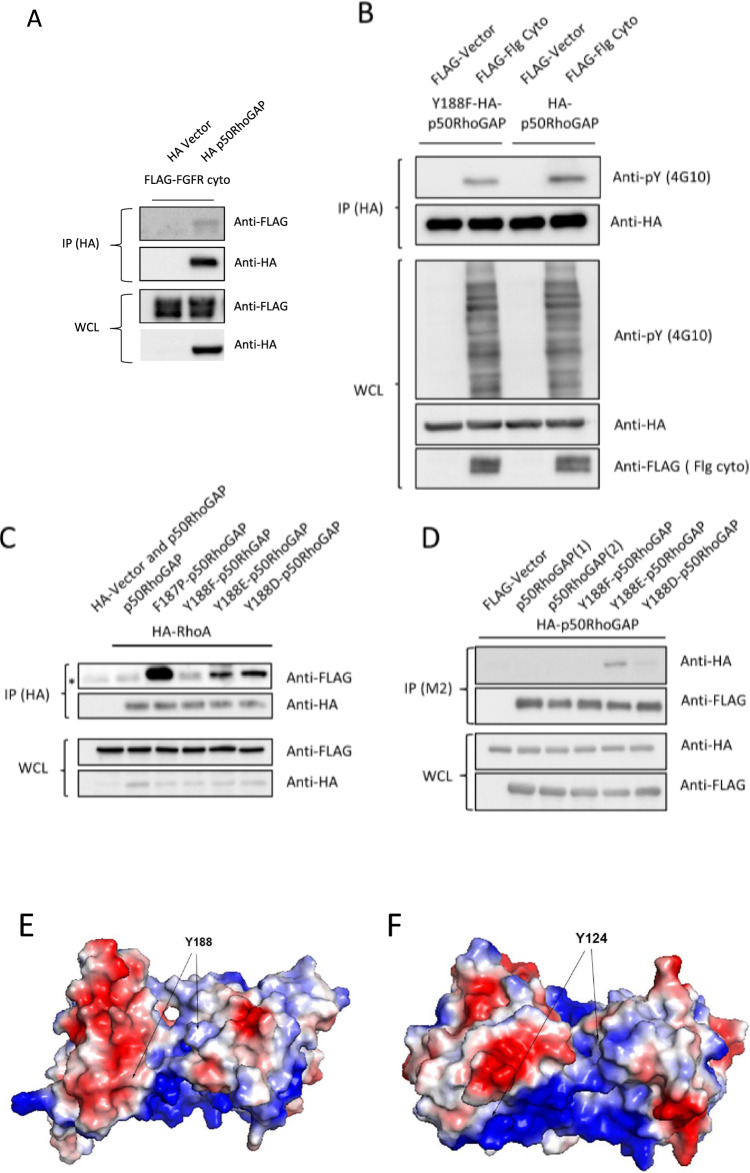
**A** Co-immunoprecipitation of FLAG-tagged Fibroblast growth factor receptor cytsolic fragment (FGFR cyto) by HA-p50RhoGAP using anti-HA magnetic beads. 293 T cells were transfected with the expression vectors FLAG-FGFR (flg) cyto and HA-vector or HA-p50RhoGAP as indicated. Bound protein complexes were resolved on SDS-PAGE and detected by the antibodies indicated. Equal loading of the lysates was demonstrated on the WCL section. **B** 293 T cells were co-transfected with FLAG-FGFR cyto or FLAG-Vector and HA-p50RhoGAP or HA-p50RhoGAP Y188F as indicated. Immunoprecipitation and SDS-PAGE/Western of the HA-tagged protein was performed and was probed with 4G10 to identify phopsho-Tyrosine signals. Anti-HA antibodies were used to demonstrate equal loading. Equal loading of the lysates was demonstrated on the WCL section. WCL was probed with 4G10 to indicate increased kinase activity by the FGFR cyto fragment. **C** 293 T cells were transfected with the expression vectors HA-RhoA and the FLAG-p50RhoGAP or its mutants as indicated. Cells were lysed and immunoprecipitated with anti-HA magnetic beads. Bound protein complexes were resolved on SDS-PAGE and detected by the antibodies indicated. Equal loading of the lysates was demonstrated on the WCL section. **D** Co-immunoprecipitation of HA-p50RhoGAP by FLAG-p50RhoGAP using anti-FLAG M2 beads. 293 T cells were transfected with the expression vectors FLAG-p50RhoGAP or its mutants and the HA-p50RhoGAP as indicated. Anti-FLAG M2 beads were used to precipitate the FLAG-p50RhoGAP and mutants. Bound protein complexes were resolved on SDS-PAGE and detected by the antibodies indicated. Equal loading of the lysates was demonstrated on the WCL section. **E** Electrostatic surface potential figure human BCH model shows that the position 188 (equivalent to 124 in yeast) is surrounded by neutral amino acids. **F** The same representation of the yBCH crystal structure shows the similar position is present in a highly basic region

We observed a highly conserved tyrosine across the BCH domains of many proteins (position 124 in _Y_BCH; 188 in _H_BCH). When we searched various disease databases, we noted a polymorphism or mutation of this residue (Y188C) in ovarian cancer [32]. We performed co-immunoprecipitation studies for WT p50RhoGAP with RhoA or p50RhoGAP itself or its mutant to see how Y188 may affect their interactions. To do that, cells were made to co-express p50RhoGAP or Y188F with a cytosolic fragment of fibroblast growth factor receptor (FGFR-cyto), a constitutively active kinase that is used only as a hypothetical kinase aimed to phosphorylate potential tyrosine residues of p50RhoGAP in order to examine its potential regulatory role (Fig. 3A). Next, we used two phosphomimic mutants, Y188E and Y188D, as well as a negative Y188F mutant designed to restrict phosphorylation. As a comparison, we also used a constitutively active F187P mutant [31] that we previously showed to disrupt the auto-inhibition of the RhoGAP domain. Collectively, we found that Y188F had reduced total phosphotyrosine signal (Fig. 3B) and there was increased binding of RhoA to Y188E-p50RhoGAP and Y188D-p50RhoGAP as compared with the WT, but with lesser extent compared to the F187P mutant (Fig. 3C). In contrast, the Y188F mutant showed very weak interaction with RhoA, similar to the WT level, indicative of the autoinhibited form of p50RhoGAP that had little or no binding to RhoA. Furthermore, the Y188E mutant, and to lesser extent with Y188D, showed increased binding to WT p50RhoGAP, suggesting that these states enhanced dimerization of p50RhoGAP compared to the WT p50RhoGAP itself (Fig. 3D). Overall, it is tempting to propose that phosphorylation at this specific tyrosine residue is linked to release of the BCH domain to enhance p50RhoGAP-p50RhoGAP interaction and for RhoA binding. Additional work is warranted to further elucidate the actual involvement of tyrosine 188 in any physiological or pathophysiological role of p50RhoGAP.

## Discussion

The p50RhoGAP (also known as Cdc42GAP) protein is widely expressed in many tissues and is responsible for controlling cell motility, morphology, polarity, and many signaling pathways. The BCH domain is identified as a distinct subclass of the Sec14p superfamily, which is known to bind to lipids and small fatty acids, but BCH domain has acquired new functional motifs [33]. The C-terminal (GTPase-activating protein) GAP domain is crucial for the regulation of many small GTPases like Rho. For p50RhoGAP, the BCH domain also maintains the GAP domain in its inactive form through autoinhibition via the β5 strand, which lies at the interface of this interaction. This auto-inhibition is crucial for the maintenance of active p50RhoGAP in the cell. Our previous structure analysis of a dimer of asymmetric monomers of the wild-type yeast BCH domain showed a sharp change in the direction of the polypeptide chain at position 116 of the BCH domain [28]. We sought to investigate the structural and functional significance of this residue at this position of p50RhoGAP.

Through sequence analysis, we identified not only a lack of conservation at this residue but also no effect to the structure or function of the protein through mutational analysis at that site. Despite the expected role of proline in changing the direction of the polypeptide chain, we surmise that position 116 has a minimal effect on the _Y_BCH domain, and that our analysis of the structure of the mutated P116A _Y_BCH suggests that this structure is representative of the BCH domain family members. Additional interrogation of the sequences, however, highlighted significant conservation of a tyrosine residue at position 188 of the human sequence, and further assessment found this residue to be mutated (_H_BCH Y188C) in ovarian cancer [32]. Interestingly, our phosphomimic mutants, Y188E and Y188D, as well as a constitutively active F187P mutant [31] showed increased RhoA binding as compared with WT. We surmise that these mutations disrupted the auto-inhibition of thep50RhoGAP.

The current findings attempt to explain the regulation of p50RhoGAP based on structural and sequence analysis, an aspect that is largely unexplored to this point. Interestingly, the conserved tyrosine at position 188 could play a crucial role in regulating the _H_BCH domain in p50RhoGAP. Through the mutations of Y188 to Y188D/E (which mimics phosphorylation), we hypothesize that this residue may undergo phosphorylation in cells, thereby modifying the charge of the region and disrupting internal auto-inhibition. Our previous studies have indicated that auto-inhibition can be released by the F187P mutation [31]. We propose that combining mutations, such as F187P/Y188E or F187P/Y188D double mutants, may further increase the population of uninhibited BCH domains compared to single mutants F187P, Y188E, or Y188D. These positions on the p50RhoGAP protein likely exert significant control over its function and thereby warrant further investigation in future studies.

In summary, our findings showed that 1) substitution of Pro116 to alanine has no effect on the scaffold of the asymmetric _Y_BCH monomers of the dimer, which retains its intertwined dimeric BCH domain structure and thus function; and 2) the conserved Tyr188 in the β5 strand of _H_BCH (Tyr124 in _Y_BCH), when substituted with acidic residues, leads to increased RhoA binding and self-dimerization, suggestive of a loss of autoinhibition of the GAP domain by this putative phosphorylation. These studies broaden our understanding of the regulatory roles of BCH domains by revealing some putative mechanisms of action that await further detailed characterization.

## Materials and methods

### Sequence analysis

The _Y_BCH sequence was taken from the SPAC1565.02c protein sequence. The BCH domains that are evolutionarily related to this p50RhoGAP protein are group III, group IIa and group IIb members [33]. 36 members were taken for this sequence comparison done on Clustal omega [36].

### Cloning, expression and purification of P116A-_YBCH_

The *S. pombe* homolog of the p50RhoGAP (_Y_BCH 1–156 a.a) was used for crystallization. Using the (His)_6_-SlyD _Y_BCH (where PP denotes the Precision Protease) construct in pET32a vector (GeneScript, Piscataway, NJ), site directed mutagenesis was carried out to substitute the Pro116 to Ala116. The parental strands were digested with DpnI and ampicillin agar plates were used to select the mutants. The mutant sequences were verified through DNA sequencing. The positive clone was transformed and grown in BL21 (DE3). The cultures were induced with 0.4 μM Isopropyl β-d-1-thiogalactopyranoside at 17 °C overnight. The cultures were pelleted and purified with Roche Ni–NTA beads with buffer A (50 mM Tris pH 7.5, 100 mM NaCl, 5% glycerol and 5 mM β-mercaptoethanol). The protein was subsequently incubated with precission protease (GE healthcare) for the cleavage of the SlyD tag and then subjected to Hi-trap SPHP (GE healthcare) cation exchange column. The mutant proteins were eluted with a gradient of buffer A and buffer B (buffer A + 1 M NaCl) followed by gel filtration of the protein with 16/60 HiLoad Superdex 200 column (GE healthcare). All purification steps were carried out at 4 °C.

### Crystallization

Crystallization screening for P116A mutant were performed with a concentration of 2.4 mg/ml using the hanging drop vapour diffusion method at room temperature (22 °C). The initially identified condition from Hampton Research (Aliso Viejo, CA) was further optimized and the best crystals were obtained from a condition consisting of 0.1 M Bis–Tris propane pH 7.0 and 2.1 M NaCl. Crystals were dehydrated in 0.1 M Bis–Tris propane pH 7.0 and 3.0 M NaCl for 1 week and cryo-protected with 25% glycerol and flash-cooled in N2 cold stream at 100 K.

### Data collection and structure determination

The data sets were collected at Advanced Photon Source (APS), USA and the National Synchrotron Radiation Research Center (NSRRC) Taiwan. The best data set was processed with HKL2000 program [37]. There were four P116A-_Y_BCH molecules in the asymmetric unit. The Matthews coefficient was estimated to be 3.9 Å^3^/Da [38], corresponding to a solvent content of 68%. The structure was solved by molecular replacement method using the wild-type structure as a search model (PDB code 8K70). The model was built using the AutoBuild program [39] followed by manual model building using COOT program [40]. The structure was refined using Phenix-refine program [41]. The refinement was done with the following parameters —Strategy: XYZ coordinates, rigid body, individual B factors and occupancies; Targets and weighing: MLHL using experimental phase restraints; other options: automatically correct N/Q/H errors with a n gaussian scattering table. The model has good stereochemistry, with 98.0% residues within the allowed regions of the Ramachandran plot analyzed by PROCHECK [42].

### Analytical ultracentrifugation

P116A (2.4 mg/ml) _Y_BCH were subjected to sedimentation velocity experiments using analytical ultracentrifugation to verify oligomerization. Sedimentation velocity profiles were collected by monitoring the absorbance at 280 nm. The samples were sedimented at 40,000 rpm at 24 °C for 5 h in a Beckman Optima XL-I centrifuge (Beckman Coulter Inc., Brea, CA) fitted with a four-hole AN-60 rotor and double-sector aluminium center pieces and equipped with absorbance optics. A total of 95 scans were collected and analysed using Sedfit.

### Site directed mutagenesis

Site-directed mutagenesis on different genes described in this paper was achieved via inverse PCR technique 50 using the Kapa HiFi DNA polymerase Kit (KAPA Biosystems, MA). Positive plasmids were verified by DNA sequencing.

### Cell culture and transfection

Human 293 T cells and HeLa cells were maintained in RPMI-1640 medium and DMEM (high glucose), respectively. Both media were supplemented with 10% (vol/vol) Fetal bovine serum, 100 U/ml penicillin & 100 mg/ml streptomycin (all from Gibco, Thermo Fisher Sci). Cells were chemically transfected with indicated plasmids expression vector t(s) using Lipofectamine 2000 (Invitrogen, Carlsbad, CA) or TransIT-LT1 (Mirus Bio, Madison, WI), according to manufacturers’ protocol.

### Construction of expression plasmids

pXJ40-tagged RhoA and p50RhoGAP expression plasmids were obtained as described in [13]. Mutants of p50RhoGAP were constructed as described in the Site-directed mutagenesis section.

### Bio-imaging

HeLa cells were cultured on coverslip and transfected with indicated plasmids. Cells were fixed with PFA and labeled with anti-FLAG (Sigma-Aldrich, St Louis, USA), anti-HA, and phallodin (Invitrogen, Thermo Fisher Sci USA). Cells were imaged by W1 spinning disk microscope (Nikon, Japan).

### Co-immunoprecipitation studies and western blot analyses

Transfected cells were lysed in modified RIPA buffer (150 mM sodium chloride, 50 mM Tris, pH 7.3, 0.25 mM EDTA, 1% sodium deoxycholate, 1% Trition-X 100, 0.2% sodium fluoride, 5 mM sodium orthovanadate, 25 mM sodium glycerophosphate and cocktail protease inhibitors (Roche Applied Science, Germany). Anti-FLAG M2 beads (Sigma-Aldrich, St Louis, USA) or Magnetic anti-HA beads (Pierce, Thermo Fisher Sci, USA) were used to immunoprecipitate FLAG-tagged or HA-tagged protein, respectively. Bound protein partners of the precipitated proteins were analyzed by western blotting. Blots were probed with anti-FLAG (Sigma-Aldrich, St Louis, USA), anti-HA (Invitrogen, USA), anti-pY antibodies 4G10-platinum (Merck Millipore, USA).

## Data Availability

Three dimensional atomic coordinates, and structure factors of _Y_BCH P116A have been deposited in the Protein Data Bank, www.pdb.org (PDB ID code 8K70).
